# Oculoplastic surgery in Madagascar: a review

**Published:** 2009-06

**Authors:** JH Norris, RP Gale, H Nkumbe, OC Backhouse, P Bernadin, BY Chang

**Affiliations:** ^1^ St James's University Hospital, Leeds, UK; ^2^ York District Hospital, York, UK; ^3^ Andranmadio Hospital, Antsirabe, Madagascar; ^4^ HJRA Government Hospital, Antananarivo, Madagascar

Oculoplastic surgery in high-income countries is now recognised as a rapidly evolving and expanding subspecialty. In low- and middle-income countries, however, most oculoplastic surgery is performed by general ophthalmologists with varying levels of surgical training and experience.

The aim of this study was to review the oculoplastic practices in Madagascar, including the nature of presenting disease and the surgical procedures performed. The study formed part of the initial phase of a training link between Madagascar and Leeds University Teaching Hospitals Trust. We hoped to identify areas of practice that required more specific surgical training. To our knowledge, this is one of the first studies looking specifically at oculoplastic disease prevalence in Madagascar or any African country.

The training link was established between Madagascar and Leeds in 2008 under the auspices of the VISION 2020 Links Programme run by the International Centre for Eye Health and supported by the Overseas Partnering and Training Initiative (OPTIN). A memorandum of understanding was signed by the Madagascar Ministry of Health, the University of Antananarivo, the Malagasy Lutheran Church Health Department, and Leeds University Teaching Hospitals Trust.

**Figure:**
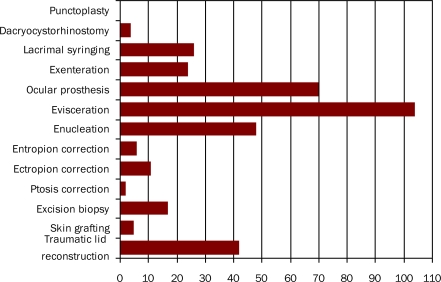
Figure 1. Oculoplastic procedures performed by fifteen Malagasy ophthalmic practitioners

**Figure:**
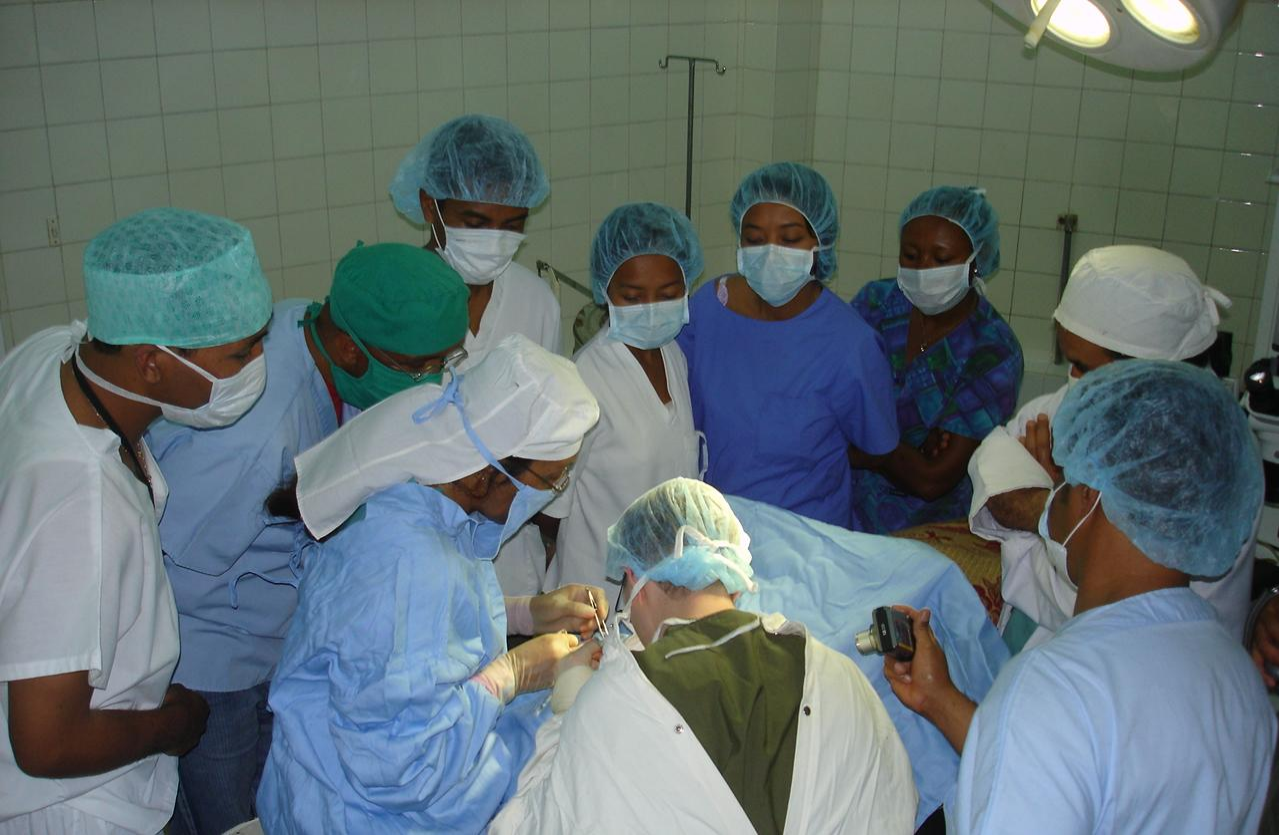
A Malagasy ophthalmologist is trained in oculoplastic surgical techniques while her team observes

Jonathan Norris

The first part of our study consisted of circulating a questionnaire to fifteen ophthalmologists from six eye departments throughout Madagascar. The questionnaire listed oculoplastic procedures related to eyelid, lacrimal, and orbital surgery and ophthalmologists were requested to indicate, from memory, the numbers of each procedure performed in the preceding eighteen months (from April 2007 to October 2008).

The practitioners reported performing 359 oculoplastic procedures in total. Eye removal, both evisceration and enucleation, accounted for 49 per cent of all procedures performed. Orbital implants (for example, coral implants) were rare due to cost. Nearly 20 per cent of procedures involved inserting a secondary conformer or prosthesis. The least common procedures included ptosis, lacrimal surgery (punctoplasty and dacryocystorhinostomy), and entropion correction; only five surgeons had used skin grafting.

Seventy five per cent of all procedures were performed by ophthalmologists based in the capital.

In the second part of the study, we surveyed all ophthalmologists and related practitioners present at the Madagascan Ophthalmology Society meeting in 2008 (n=33) about surgical interventions for ectropion, entropion, and ptosis.

Of the ophthalmic practitioners who had reviewed ectropion, only 16.6 per cent had performed corrective surgery. Of those who had reviewed entropion and ptosis, 22.7 per cent and 14.2 per cent, respectively, had performed corrective surgery (Figure 2).

For the third part of the study, we reviewed 23 patients who had been collected for us to see in a single day at a government hospital in Antananarivo, the capital of Madagascar (the tertiary ophthalmic referral centre in the country). Table 1 shows the pathology seen. We performed surgery on nine of the patients. Procedures included traumatic lid reconstruction surgery (with free grafts, transposition flaps, and Z-plasty), prolene brow suspensions, upper lid ectropion correction with anterior lamellar repositioning, and lower lid entropion correction and orbital dermis fat graft for postenucleation socket syndrome (PESS). During each procedure, we gave specific training to the local ophthalmic practitioners present. Malagasy ophthalmic practitioners have now begun to perform some of the above procedures.

**Figure:**
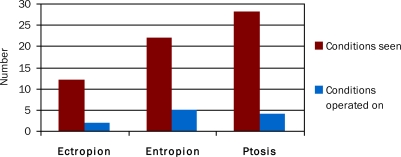
Figure 2. The number of conditions seen compared with those operated on in the previous twelve months

## Discussion

Madagascan ophthalmic practitioners centre their attention on enucleation, evisceration, and exenteration for presumed traumatic and neoplastic causes. Previous studies have indicated that the most common causes for eye removal in African countries is both trauma and orbito-ocular tumours,^1^ including retinoblastoma and Burkitts lymphoma.^2^ Squamous cell carcinoma also accounts for a significant amount of orbito-ocular neoplastic disease in certain African countries.^3^ Infection would be considered another leading cause.^4^ Incidentally, when performing two orbital dermis fat grafts for socket atrophy on patients at the government hospital, we found residual uveal tissue in both cases, giving a theoretical higher risk of sympathetic ophthalmia.^5^

Patients in Madagascar are more susceptible to post-enucleation socket syndrome (PESS) because of the lack of orbital implants. Training in both eye removal and orbital dermis fat grafting should help improve practice in the future.

Given the limited resources, lacrimal surgery is rarely performed, presumably because lacrimal pathology is rarely life or sight threatening. Ectropion, entropion, and ptosis are present in Madagascar, but few are corrected surgically. The results for entropion surgery (22.7 per cent) are similar to those for bilamellar tarsal rotation for trichiasis (24.6 per cent) in a study of leprosy patients in Nigeria.^6^ In our study, the low surgical coverage was felt to be mainly due to lack of patient awareness. We believe a lack of specific surgical training should also be considered a factor.

We understand that this three-part study was limited by the fact that some of the data obtained regarding oculoplastic procedures was from the practitioner's memory as opposed to a formal log book. However, we feel that we have gained a broad understanding of the current oculoplastic practice in terms of both quantity and breadth of surgical technique. This will allow us to plan future visits and target specific training needs.

**Table 1:** Presentations seen in a designated outpatient oculoplastic clinic

Case	Age (Years)	Presentation
1	12	Congenital ptosis
2	8	Congenital ptosis
3	30	Malignant melanoma of lower eyelid
4	13	Stevens Johnson syndrome with secondary cicatricial eyelid disease
5	9 months	Congenital ectropion
6	Unknown	Ptosis
7	50	Traumatic eyelid injury
8	22	Previous excision of lacrimal gland lesion and secondary ptosis
9	11	Congenital lid lesion
10	60	Ectropion
11	Unknown	Stevens Johnson syndrome with lower eyelid cicatricial, forniceal shortening
12	30	Traumatic ectropion
13	34	Conjunctival limbal lesion
14	12	Post enucleation socket syndrome
15	Unknown	Ocular dermoid cyst
16	31	Arterio-venous malformations of eyelid
17	21	Traumatic eyelid injury with lagophthalmos, ectropion, exposure keratopathy
18	86	Entropion
19	52	Entropion
20	37	Traumatic lagophthalmos
21	25	Post enucleation socket syndrome
22	33	Entropion secondary to leprosy
23	26	Orbital gunshot injury
